# Analysis of the concurrent validity and reliability of five common clinical goniometric devices

**DOI:** 10.1038/s41598-023-48344-6

**Published:** 2023-11-27

**Authors:** Sirirat Kiatkulanusorn, Nongnuch Luangpon, Wirasinee Srijunto, Sarawoot Watechagit, Krittipat Pitchayadejanant, Sireetorn Kuharat, O. Anwar Bég, Bhornluck Paepetch Suato

**Affiliations:** 1 Department of Physical Therapy, Faculty of Allied Health Sciences, Burapha University, Chonburi, 20131 Thailand; 2 Faculty of Engineering, Mahidol University, Phutthamonthon, Nakhon Pathom, Thailand; 3 Burapha University International College, Burapha University, Chonburi, 20131 Thailand; 4 Corrosion Lab, Multi-Physical Engineering Sciences Group (MPESG), Department of Mechanical/Aeronautical Engineering, Salford University, 3-08, SEE Building, Manchester, M54WT UK

**Keywords:** Physical examination, Biomedical engineering

## Abstract

Measurement errors play an important role in the development of goniometric equipment, devices used to measure range of motion. Reasonable validity and reliability are critical for both the device and examiner before and after to testing in human subjects. The objective is to evaluate the concurrent validity and reliability of five different clinical goniometric devices for the purpose of establishing an acceptable measurement error margin for a novel device. We explored the validity and inter- and intrarater reliability scores of five goniometric devices namely (i) the universal goniometer (UG), a two-armed hand-held goniometer, (ii) the inclinometer (IC), featuring a single base, fluid level, and gravity-weighted inclinometer, (iii) the digital inclinometer (DI), functioning as both a DI and dynamometer, (iv) the smartphone application (SA), employing gyroscope-based technology within a smartphone platform application and (v) the modified inclinometer (MI), a gravity pendulum-based inclinometer equipped with a specialized fixing apparatus. Measurements were obtained at 12 standard angles and 8 human shoulder flexion angles ranging from 0° to 180°. Over two testing sessions, 120 standardized angle measurements and 160 shoulder angle measurements from 20 shoulders were repetitively taken by three examiners for each device. The intraclass correlation coefficient (ICC), standard error of measurement (SEM), and minimal detectable change (MDC) were calculated to assess reliability and validity. Concurrent validity was also evaluated through the execution of the 95% limit of agreement (95% LOA) and Bland–Altman plots, with comparisons made to the UG. The concurrent validity for all device pairs was excellent in both study phases (ICC > 0.99, 95% LOA − 4.11° to 4.04° for standard angles, and − 10.98° to 11.36° for human joint angles). Inter- and intrarater reliability scores for standard angles were excellent across all devices (ICC > 0.98, SEM 0.59°–1.75°, MDC 1°–4°), with DI showing superior reliability. For human joint angles, device reliability ranged from moderate to excellent (ICC 0.697–0.975, SEM 1.93°–4.64°, MDC 5°–11° for inter-rater reliability; ICC 0.660–0.996, SEM 0.77°–4.06°, MDC 2°–9° for intra-rater reliability), with SA demonstrating superior reliability. Wider angle measurement however resulted in reduced device reliability. In conclusion, our study demonstrates that it is essential to assess measurement errors independently for standard and human joint angles. The DI is the preferred reference for standard angle testing, while the SA is recommended for human joint angle testing. Separate evaluations across the complete 0°–180° range offer valuable insights.

## Introduction

Goniometry employs various measurement tools for the clinical assessment of range of motion (ROM), a fundamental parameter used to evaluate the functionality of human joint movement and mobility^1,2^. It is essential for diagnosing pathology, monitoring pathology progression, and predicting prognosis in terms of orthopedics and rehabilitation but is not limited to athletic performance^3,4^. Therefore, goniometric devices must exhibit high validity and reliability with minimal error, and the technique for using these devices should be easily reproducible^5^. Unfortunately, obtaining precise and consistent ROM measurements has been extremely difficult owing to the considerable complexity of the anatomy and associated movements^6^. Subsequently, many tools have been developed to measure mobility, ranging from simple visual examination to complex three-dimensional mobility assessment, in order to support the overarching goals of the Sustainable Development Goals, particularly in the areas of Good Health and Well-being. Over time, the development of medical tools for ROM measurement has proceeded in parallel with general developments in technology; examples include the electrogoniometer^7^; goniometers with short or long arms^8^; laser projection with a Halo digital goniometer (laser projection used as a goniometric arm)^9^; photogrammetry software^10^; digital goniometers^11^; the Hawk goniometer, a digital-based goniometer with a plastic, parallel-piped sensor and internal gyroscope^12^; inertial sensors for real-time monitoring^13^; and smartphone applications (SA), which employs an inertial measurement unit (IMU)-based goniometer^14,15^. However, any technology that does not provide valid and reliable measurements is not a suitable basis for clinical decisions. Moreover, portability, cost, convenience, and suitability for everyday rehabilitation practice remain a gap for further development of goniometric devices.

To be clinically useful, ROM measurement tools must be confirmed for validity and reliability. Studies on the reliability of equipment for measuring ROM^16^ have shown the influence of instrumentation, procedures, discrepancy of movement direction, distinction of body parts and different patient types. Peters et al.^17^ found inconsistency in the reliability of goniometric devices for assessing ROM from one clinician to another; validity and reliability can be impacted by irregularities during measurement—for example, bony landmark positioning, accuracy, consistency of the examiner in establishing the zero point and positioning of the instrument against the target body segment^16,18^, may all contribute to increasing the risk of error. Human soft tissue and the inability to “see” joint centers and bone are aspects that must be considered in addition to examining the measurement properties of goniometric devices. These significantly impact examiner factors. Obviously, ROM measurement errors stem from three sources: the device, the examiner, and the patient^19,20^. Ideally, validity and reliability should be transparently investigated. Errors emerging from the equipment should be minimized during the development process.

In the process of developing a ROM measurement device, it is essential to address two of the three primary sources of variability^19^. First one must address variability inherent in the capacity of the device to quantify angular differences. Second one must accommodate variability arising from the examiner’s skill in using a device to measure angles. Thereafter, human-specific factors will also contribute to measurement variability. A previous study addressed this point by using standard angles to account for the human variability factor, namely Carvalho et al.^10^. They examined the reliability and reproducibility of goniometric measurements, compared with hand photogrammetry, by using standard angles with a wax hand mold. Volunteer examiners were instructed to position the fulcrum of the goniometer, corresponding to the axes of each joint, according to their clinical experience; then, photographic records were taken for analysis. Wellmon et al.^19^ examined the concurrent validity and interrater reliability of two goniometer mobile applications, the inclinometer (IC), and universal goniometer (UG)—by applying standardized angles from wooden models. This effectively fixed patient factors that can affect repeated measurements, enabling examination of concurrent validity and interrater reliability relating to examiner skill and the accuracy of smartphone devices and applications for determining angular excursion. Unfortunately, the acceptable degree of reliability exhibited by the equipment and examiner without patient factors has not been clearly described in the literature. This gap subsequently influences the success of the invention of a new goniometric device by limiting the inventor’s ability to proceed to the next step of conducting a study on human joints.

An in-depth analysis of measurement error, originating from the precision of equipment and the expertise of examiners, in the context of both standard joint assessments and human joint measurements, is imperative. Drawing upon the knowledge gained from widely adopted clinical instruments or gold standard can provide valuable guidance for the development of new measurement tools^5^. Although radiographic measurement has been acknowledged as the gold standard^21^, it results in unnecessary radiation exposure and cannot necessarily be used reliably to measure changes in ROM^9^. Meanwhile, UG and IC have been most extensively implemented in clinical settings since the past because of their portability, low cost, convenience, and reasonable validity and reliability^22–24^. Numerous studies have indicated that the intra- and interrater reliabilities of the UG in the assessment of human joint ROM were excellent, with intraclass correlation coefficient (ICC) values consistently exceeding 0.90^8,23,24^. The certainty of the application of UG to clinical practice was reinforced by the fact that the validity of UG compared with that of radiographic measurements was high, as indicated by an ICC value of > 0.90^23,24^. Although the reliability and validity of IC for measuring human joint ROM varied from poor to excellent, it has been widely used to measure spinal ROM^25,26^. The digital inclinometer (DI) is portable, accurate, and reliable; therefore, theoretically, it can be applied in practice^18,27,28^; however, it comes at a higher cost than both the UG and IC^29^. Recently, our approach to patient management in rehabilitation practice has evolved due to the impact of novel technologies and the use of computer-based applications (apps). A recent systematic review of the validity and reliability of SAs for ROM measurement has sufficiently supported their viability as goniometer substitutes^14^. Because the IC can be modified (modified inclinometer [MI]) by attaching a fixing apparatus to free the examiner’s hand, reading the scale, stabilizing the extremity, and guiding movement can be accomplished by one examiner^30^. MI has been used in a particular rehabilitation approach; however, its validity and reliability have been strongly confirmed. In conclusion, the UG, IC, SA, DI, and MI have gained popularity in clinical settings because of their ease of access and compatibility in terms of size and weight, making them convenient choices for diverse applications across different settings. However, note that each device comes with its unique set of advantages and disadvantages, which has led to their selection in different settings (Table 1). Therefore, an analysis of the validity and reliability of these different angular measurement devices constitutes a priority research gap that should be addressed to determine the inherent technical error, which should be taken as a reference while developing any given new device.

**Table 1:** Comparative analysis of the characteristics of five common clinical goniometric devices.

Review criteria	Universal goniometer	Inclinometer	Smartphone application	Digital inclinometer	Modified inclinometer
Portability	Yes	Yes	Yes	Ambiguous	Yes
Ease of use	Yes	Yes	Yes	Yes	Yes
Accessibility	Yes	Yes	Yes	Ambiguous	Ambiguous
Scale-free	No	No	Yes	Yes	No
Weight	0.3 lbs	0.3 lb	Smartphone weight	4 lb	Slightly over the original IC
Dimension	12.5′′ × 0.2′′ × 5′′	4′′ × 0.2′′ × 4′′	Smartphone dimension	13′′ × 9′′ × 8′′	Similar to the original IC
Even price	$18.00	$77.50	Smartphone price	$1,723.05	Ambiguous

This study aimed to explore the concurrent validity and intra- and interrater reliabilities of five goniometric devices (i.e., UG, IC, SA, DI, and MI) by focusing on examiner factors and the measurement error of the devices. This study was conducted to provide valuable insights into setting thresholds for measurement error to help inventors and practitioners determine the suitability of new devices for joint angle measurements in human subjects.

## Methods

### Design

This study employed a descriptive, non-experimental study design. Concurrent validity and reliability were evaluated using the test/re–test method. Five common clinical goniometric devices (i.e., UG, IC, DI, SA, and MI) were employed to measure standardized ROM (ranging from 0° to 180°) and human shoulder joint flexion angles (ranging from 0° to 180°). These measurements were taken by three examiners during two testing sessions. In the concurrent validity study, the UG was selected as the reference standard for comparison because of its widespread use in clinical practice, aligning with the common practice in similar studies^18,19,31^.

### Raters and samples

**Figure 1:**
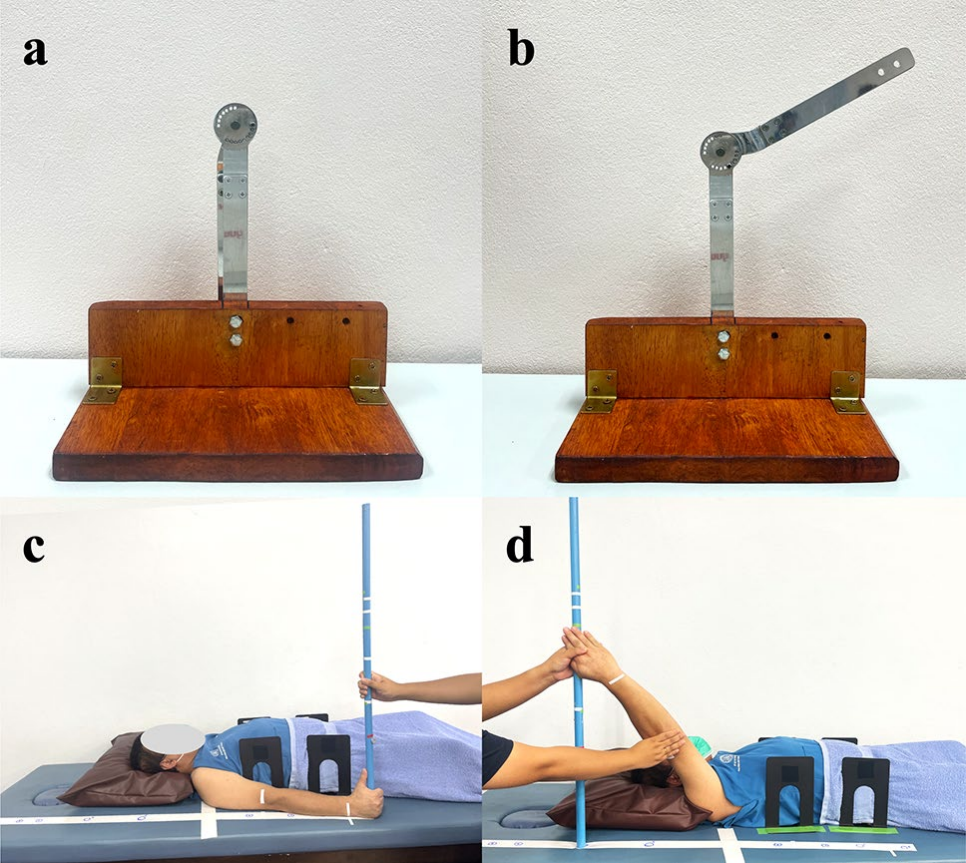
Example of standard measurement angles and human shoulder flexion angles. Starting and final measurement positions for: (**a** and **b**) standard measurement angle; (**c** and **d**) human shoulder flexion angle.

All three examiners were physical therapists with > 10 years of experience. To standardize the angles measured in the test/re–retest, a testing apparatus was developed to simulate the movement of the shoulder joint, which has the largest arc of movement among human joints (Fig. 1). The apparatus consisted of two arms joined together at one end for the axis of movement. The first arm was slightly curved, mimicking the humerus. The second arm was a straight, stationary arm fixed at one end to the wooden base. The axial end of the straight arm held a circular fitting with 16 holes used to fix the two arms in relation to a specific measurement angle. Twelve angles were set, ranging from 0° to 180°. Each angle was measured for 10 trials; thus, there were 120 measurements in total for each examiner with each device.

During the human joint angle measurement phase, measurements were taken from a group of 20 healthy shoulders, consisting of 10 individuals (5 males and 5 females) with an average age of 23.10 ± 3.25 years, an average weight of 68.70 ± 21.33 kg, an average height of 166.60 ± 6.88 cm and an average body mass index of 24.74 ± 7.49 kg/m^2^. Each shoulder was assessed at 8 different angles, ranging from 0° to 180°. This resulted in 160 measurements for each examiner using each device.

To assess reliability, measurements of each of the three standardized angles and each of the two shoulder flexion angles were analyzed, ensuring that at least 30 heterogeneous samples were examined^32^. Groups of three sequences of standardized angles and groups of two sequences of human shoulder flexion angle lying in the same quarter of the semicircle were analyzed, as follows: 1st quarter, 0°–45°; 2nd quarter, > 45°–90°; 3rd quarter, > 90°–135°; 4th quarter, > 135°–180°.

### Procedures

The same evaluation conditions were maintained for each examiner at each testing session, encompassing both study phases. Before data collection for each phase, all examiners participated in a practice session to clarify the study procedure and measurement methods for all devices. Three examiners (Researchers B.S, N.L., and W.S.) measured the standardized angles and human shoulder joint flexion angles using each device (i.e., UG, IC, DI, SA, and MI) in a random order. Each standardized angle and each shoulder flexion angle of every participant underwent multiple measurements by each examiner using every designated device. The measurements were performed in two testing sessions, with a 2-week gap between sessions for standardized angle measurements and a 2-day gap for shoulder flexion angle measurements. The assignment and order of the 12 standardized angles and 8 shoulder flexion angles for each participant were randomly determined by Researcher S.W. To blind the examiners to the data recorded, readings were taken by a second investigator (Researcher S.K.) and recorded by an assistant researcher. Whole numbers at 1° increments were recorded.

The process of establishing shoulder flexion angles for all participants was meticulously performed while they were in the supine position (lying face upwards). To maintain precision and consistency in the starting position for each testing instance, markers were strategically placed to delineate the positions of the entire trunk and the testing arm on the bed. This careful approach was instrumental in achieving a uniform starting point for all measurements. Shoulder flexion angles were systematically determined using a polyvinyl chloride (PVC) pipe that featured distinctive markings on both the PVC pipe itself and the bed. This standardization process was diligently supervised by the same assistant researcher for all participants, ensuring that the angle settings were accurate and consistent across the board. During the human testing phase, the specific shoulder flexion angle was meticulously set by the designated assistant researcher. Subsequently, the examiner responsible for the final adjustments and alignment played a critical role in ensuring that the measurement device was precisely positioned before making measurements.

### Goniometric measurements

**Figure 2:**
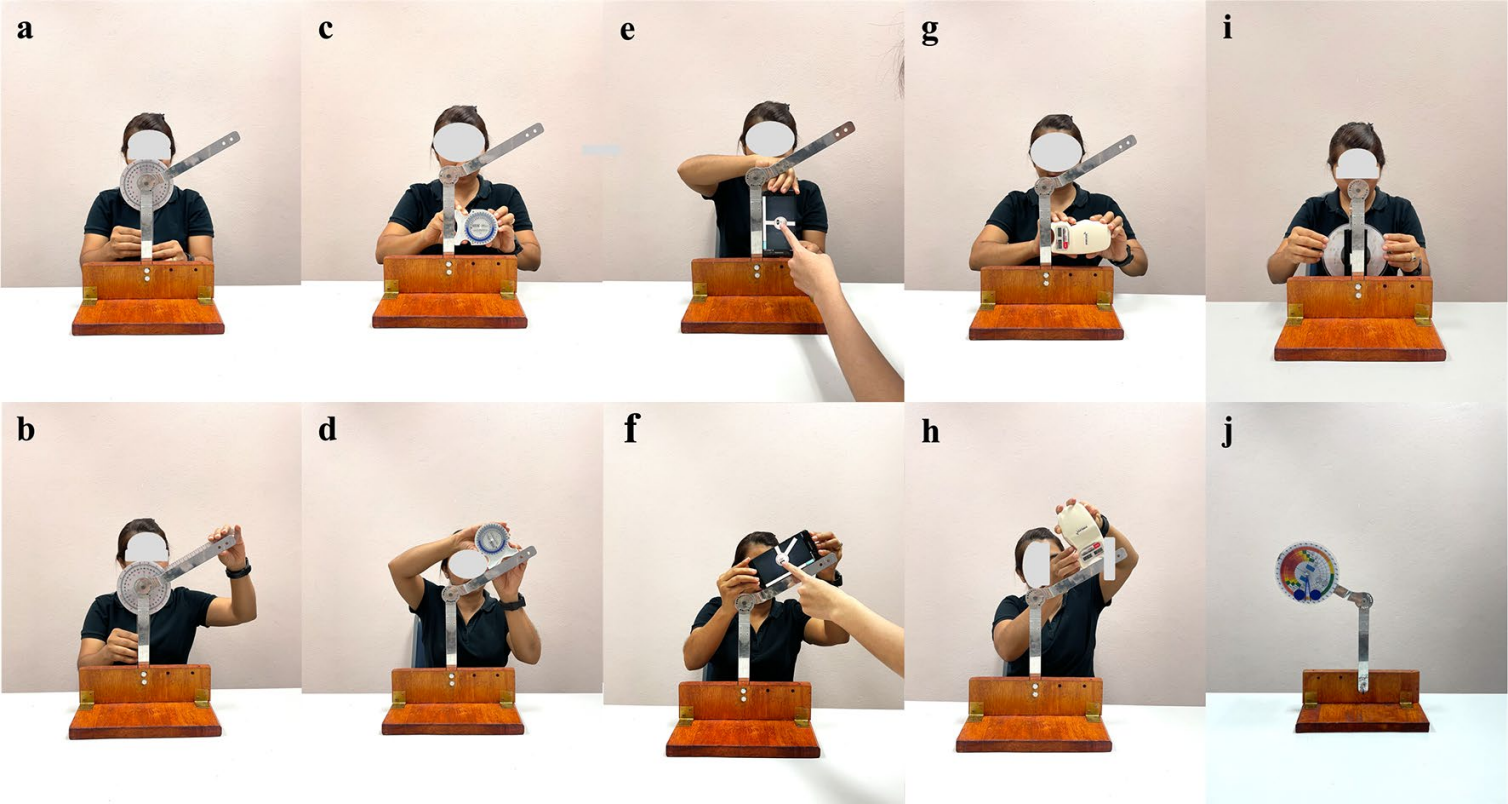
Goniometric devices and procedures for measuring standard angle. Starting and final measurement positions for: (**a** and **b**) universal goniometer; (**c** and **d**) inclinometer; (**e** and **f**) smartphone application; (**g** and **h**) digital inclinometer; (**i** and **j**) modified inclinometer.

**Figure 3:**
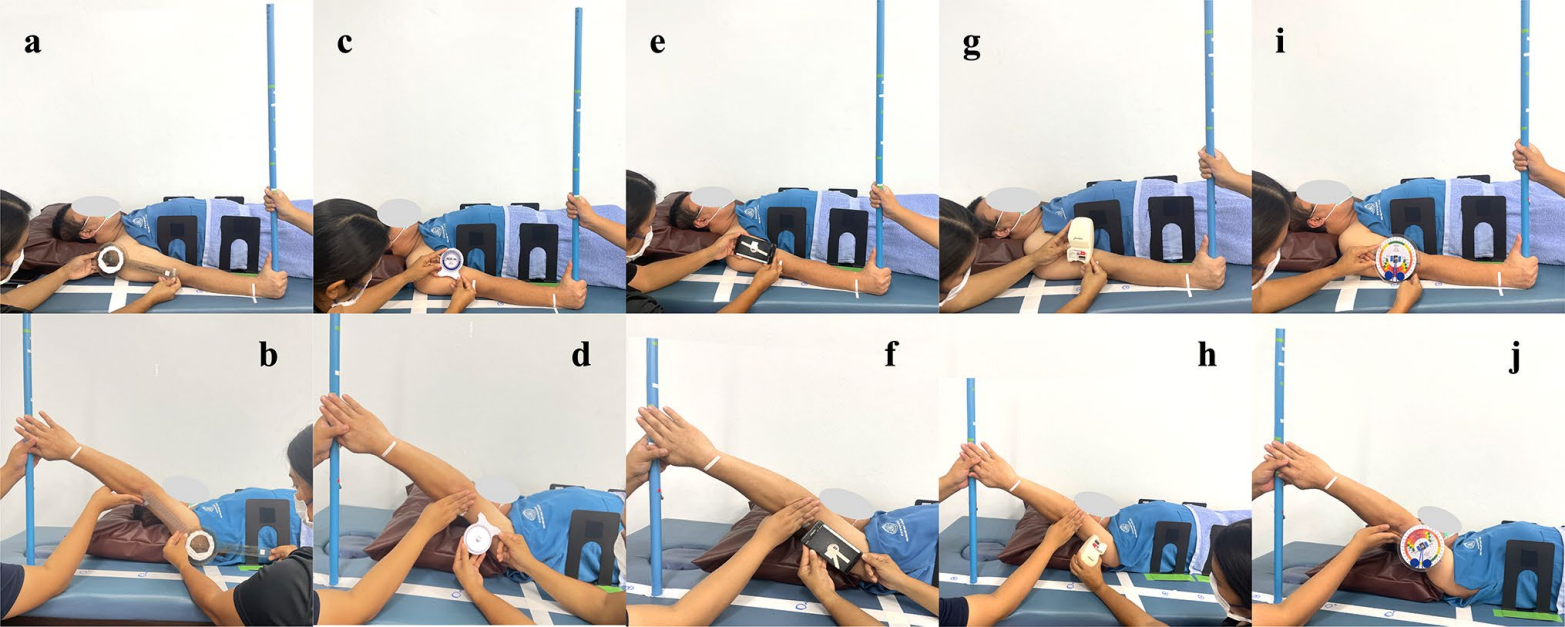
Goniometric devices and procedures for measuring human shoulder flexion angle. Starting and final measurement positions for: (**a** and **b**) universal goniometer; (**c** and **d**) inclinometer; (**e** and **f**) smartphone application; (**g** and **h**) digital inclinometer; (**i** and **j**) modified inclinometer.

Figures 2 and 3 shows the measurement procedures used for all goniometric devices. To blind the examiner to the readings, the scale, screen or monitor of each device was directed away or covered.

The UG used in this study was a 12-inch transparent plastic model, specifically the Baseline® Model 12-1000 (*Fabrication Enterprises, White Plains, NY, USA)*, featuring a protractor scale, two arms, and a fulcrum. The IC used was a 180° Baseline Bubble® (Fabrication Enterprises), which operates based on fluid levels. These two devices present a 360° scale with 1° increments. To measure angles using the UG, the examiners positioned the fulcrum of the UG on the axis of the apparatus (Fig. 2a and b) or acromion process of the participant’s shoulder and aligned the UG’s stationary and movable arms to the arms of the apparatus or the participant’s humerus and trunk (Fig. 3a and b). For the IC, the examiners positioned the base of the IC against the two arms of the apparatus (Fig. 2c and d) or the participant’s humerus in two consecutive positions (Fig. 3c and d).

The gyroscope-based goniometer was a Samsung Galaxy Note Fan edition smartphone running the Goniometer Records application (Indian Orthopedic Research Group, www.iorg.co.in/2013/05/goniometer-records-mobile-app/). This application was chosen because it is free on Google Play and quite accurate^19,33^. During the measurement process, the alignment of the smartphone’s edge with either the arms of the apparatus (Fig. 2e and f) or the participant’s humerus was performed in two consecutive steps (Fig. 3e and f).

In this study, the MicroFET® 3 DI (*Hoggan Scientific in Salt Lake City, UT, USA)* was used. This device was chosen for its versatility, as it can serve as both a handheld dynamometer and a DI. It is known for its cost-effectiveness and ease of implementation in a clinical setting^34^. For measurements, the examiner placed the device parallel to the stationary arm of the apparatus or the participant’s humerus at the starting position. The reading angle was recorded when the examiner aligned the device with the movable arm of the apparatus (Fig. 2g and h) or the participant’s humerus at the final position and pressed the “Final Setting” button on the side of the device (Fig. 3g and h).

In this study, the MI employed was a gravity pendulum-based IC originally designed as a low-cost goniometer. Modifications were made to this device, including the addition of an adjustable scale and a gravity pendulum reading scale. Furthermore, a fixing apparatus was used with the inclinometer. This design was proposed in order to free the examiner’s hands for controlling unwanted movements during ROM measurements. During measurement, the device was attached to the patient, allowing the examiner to use their hands to support the patient’s movement. To measure the sample angles in this study, the examiner fixed the device to the movable arm of the apparatus or the participant’s arm and set the zero scale when the movable arm remained in its starting position. To ensure that the examiner was blinded, the readings were observed and recorded by a second investigator as the movable arm of the apparatus (Fig. 2i and j) or the participant’s arm moved into the final position (Fig. 3i and j).

### Statistical analysis

Descriptive statistics of the 12 standardized angles and 8 human joint angles measured by all examiners using all devices in both testing sessions were calculated. The ICC values of the two-way mixed model were calculated to describe concurrent validity and inter- and intrarater reliabilities. These analyses were performed separately for the two study phases: standard angle measurement and human joint angle measurement. Inter- and intrarater reliabilities were considered in terms of the ICC as follows: poor, < 0.5; moderate, 0.5–0.75; good, 0.75–0.9, and excellent, > 0.9^35^. As an additional examination of concurrent validity and reliability, the standard error of measurement (SEM) was calculated in relation to the ICC using the following formula: SEM = standard deviation (SD) × √(1 − r)^35,36^. The SEM is often employed for clinical measurement procedures to avoid intersample variability^37^. A lower SEM implies greater measurement accuracy. To determine the true changes in ROM (*vs.* random error), the minimal detectable change (MDC) at the 90% confidence level was calculated using the following formula: MDC = 1.65 × SEM × √2^37^. To reflect the smallest unit of measurement of all goniometric devices, the MDC values were rounded to the nearest degree. The concurrent validity between two measurement devices was described as reasonable validity when the ICC was > 0.90^35^. Furthermore, agreement and systematic differences between measurement devices were examined using Bland–Altman plots. Differences relative to the range of true measurements were assessed using 95% limits of agreement (95% LOA), calculated as follows: mean difference between devices ± 1.96 × SD^35,38^.

### Ethics approval and consent to participate

All participants signed a consent form before testing. The study was conducted according to the Declaration of Helsinki and was approved by the Ethics Committee of Burapha University under protocol number HS014/2566(C1) and IRB number IRB1-070/2566.

## Results

Tables 2 and 3 show descriptive data for each standardized angle and human joint angle, measured using all five devices (i.e., UG, IG, SA, DI, and MI) during both testing sessions. No significant differences in ROM measurements were observed between raters (F = 0.086, *P* = 0.918, ES < 0.001; F = 0.142, *P* = 0.868, ES < 0.001), between devices (F = 0.055, P = 0.994, ES < 0.001; F = 0.232, *P* = 0.921, ES < 0.001), and between testing sessions (F = 0.091, *P* = 0.764, ES < 0.001; F = 0.188, *P* = 0.664, ES < 0.001) for both standardized angle and human joint angle measurements.

**Table 2:** Mean and standard deviation (SD) of the angle measured by five goniometric devices.

Measure angle	Testing session	Mean ± SD				
		Universal goniometer (n = 30)	Inclinometer (n = 30)	Smartphone application (n = 30)	Digital inclinometer (n = 30)	Modified inclinometer (n = 30)
1	1st	11.77 ± 0.82	12.07 ± 0.78	11.30 ± 0.53	11.57 ± 0.57	12.77 ± 0.97
	2nd	11.33 ± 0.61	11.47 ± 0.63	10.93 ± 0.37	11.20 ± 0.55	11.27 ± 0.64
2	1st	28.73 ± 0.64	28.80 ± 0.76	28.10 ± 0.48	28.40 ± 0.62	29.07 ± 1.39
	2nd	28.43 ± 0.50	27.67 ± 0.55	27.37 ± 0.61	27.23 ± 0.82	28.47 ± 0.68
3	1st	41.97 ± 0.76	42.13 ± 0.68	41.23 ± 0.50	41.87 ± 0.51	40.67 ± 1.15
	2nd	41.53 ± 0.78	41.23 ± 0.68	40.60 ± 0.67	40.93 ± 0.52	40.57 ± 0.73
4	1st	60.77 ± 0.94	60.00 ± 0.83	58.43 ± 0.57	59.60 ± 0.72	60.57 ± 0.94
	2nd	59.53 ± 0.63	59.83 ± 0.75	58.37 ± 0.61	59.20 ± 0.48	59.30 ± 1.95
5	1st	74.47 ± 0.90	74.33 ± 0.71	73.20 ± 1.16	74.20 ± 0.76	73.93 ± 2.18
	2nd	74.63 ± 0.49	74.13 ± 0.43	73.13 ± 0.63	74.40 ± 0.50	73.73 ± 0.78
6	1st	88.87 ± 1.17	89.13 ± 1.46	87.57 ± 0.9	88.5 ± 0.51	89.67 ± 1.37
	2nd	89.53 ± 0.68	88.8 ± 0.76	87.9 ± 1.88	88.87 ± 0.35	88.6 ± 0.62
7	1st	103.63 ± 1.59	104.23 ± 0.90	103.73 ± 0.83	103.83 ± 0.70	105.27 ± 1.08
	2nd	103.87 ± 0.73	103.63 ± 0.96	102.77 ± 1.91	103.67 ± 0.80	103.93 ± 0.83
8	1st	117.07 ± 1.62	118.40 ± 1.63	117.73 ± 0.87	117.83 ± 0.95	119.57 ± 1.98
	2nd	118.53 ± 0.86	118.57 ± 0.68	117.83 ± 1.70	118.27 ± 0.52	117.73 ± 0.78
9	1st	133.87 ± 0.97	134.40 ± 0.97	133.53 ± 0.82	133.73 ± 0.78	134.07 ± 1.84
	2nd	133.60 ± 0.62	133.87 ± 1.22	132.77 ± 2.16	134.10 ± 1.32	134.77 ± 1.01
10	1st	148.83 ± 1.60	148.67 ± 1.15	148.23 ± 1.50	147.47 ± 0.68	148.80 ± 1.40
	2nd	147.97 ± 0.72	149.03 ± 0.72	146.50 ± 1.59	147.43 ± 0.86	147.40 ± 1.16
11	1st	165.80 ± 2.09	165.30 ± 1.53	164.17 ± 1.15	164.20 ± 1.06	165.30 ± 1.99
	2nd	164.07 ± 0.83	165.43 ± 0.97	162.03 ± 1.81	163.30 ± 0.60	163.97 ± 0.96
12	1st	179.37 ± 0.93	179.60 ± 1.16	178.27 ± 1.01	178.07 ± 1.23	179.43 ± 1.87
	2nd	178.83 ± 0.70	178.43 ± 0.94	177.50 ± 1.50	176.93 ± 0.83	177.57 ± 1.17

**Table 3:** Mean and standard deviation (SD) of human shoulder range of motion (ROM) measured using five goniometric devices.

Measure angle	Testing session	Mean ± SD				
		Universal goniometer (n = 40)	Inclinometer (n = 40)	Smartphone application (n = 40)	Digital inclinometer (n = 40)	Modified inclinometer (n = 40)
1	1st	17.25 ± 2.38	17.65 ± 2.36	17.40 ± 2.37	17.33 ± 2.18	17.12 ± 3.23
	2nd	16.82 ± 2.08	17.07 ± 1.99	16.72 ± 2.22	16.35 ± 2.48	16.75 ± 2.69
2	1st	39.38 ± 3.41	41.78 ± 2.72	40.70 ± 3.22	41.22 ± 2.38	40.37 ± 3.83
	2nd	40.48 ± 3.11	40.98 ± 2.63	40.87 ± 2.76	41.33 ± 3.06	40.75 ± 3.48
3	1st	59.15 ± 3.95	62.02 ± 2.84	60.23 ± 2.93	61.48 ± 2.83	60.32 ± 4.18
	2nd	59.07 ± 3.10	61.48 ± 3.49	59.42 ± 3.06	60.70 ± 3.01	59.50 ± 3.54
4	1st	82.00 ± 3.58	86.18 ± 3.32	84.37 ± 3.28	86.17 ± 3.60	83.52 ± 4.03
	2nd	82.63 ± 2.91	84.93 ± 2.15	83.60 ± 2.78	84.98 ± 2.88	83.62 ± 3.07
5	1st	107.72 ± 3.88	109.18 ± 4.85	109.23 ± 4.70	108.07 ± 3.40	104.03 ± 3.63
	2nd	106.3 ± 3.06	107.02 ± 4.09	108.40 ± 3.53	106.87 ± 2.85	106.05 ± 4.52
6	1st	131.62 ± 4.31	134.80 ± 5.94	133.42 ± 5.22	132.03 ± 4.28	130.45 ± 5.53
	2nd	130.25 ± 2.89	131.77 ± 3.21	132.67 ± 3.93	130.95 ± 3.28	131.58 ± 3.85
7	1st	145.78 ± 4.72	147.25 ± 5.43	146.70 ± 5.13	148.32 ± 5.69	145.03 ± 4.14
	2nd	145.10 ± 3.13	145.47 ± 4.12	147.00 ± 4.64	146.62 ± 4.33	146.08 ± 4.39
8	1st	156.68 ± 5.22	157.80 ± 6.15	157.50 ± 5.66	160.27 ± 8.54	156.05 ± 4.11
	2nd	155.48 ± 3.63	155.73 ± 4.87	157.38 ± 5.36	156.93 ± 4.86	157.73 ± 5.49

**Figure 4:**
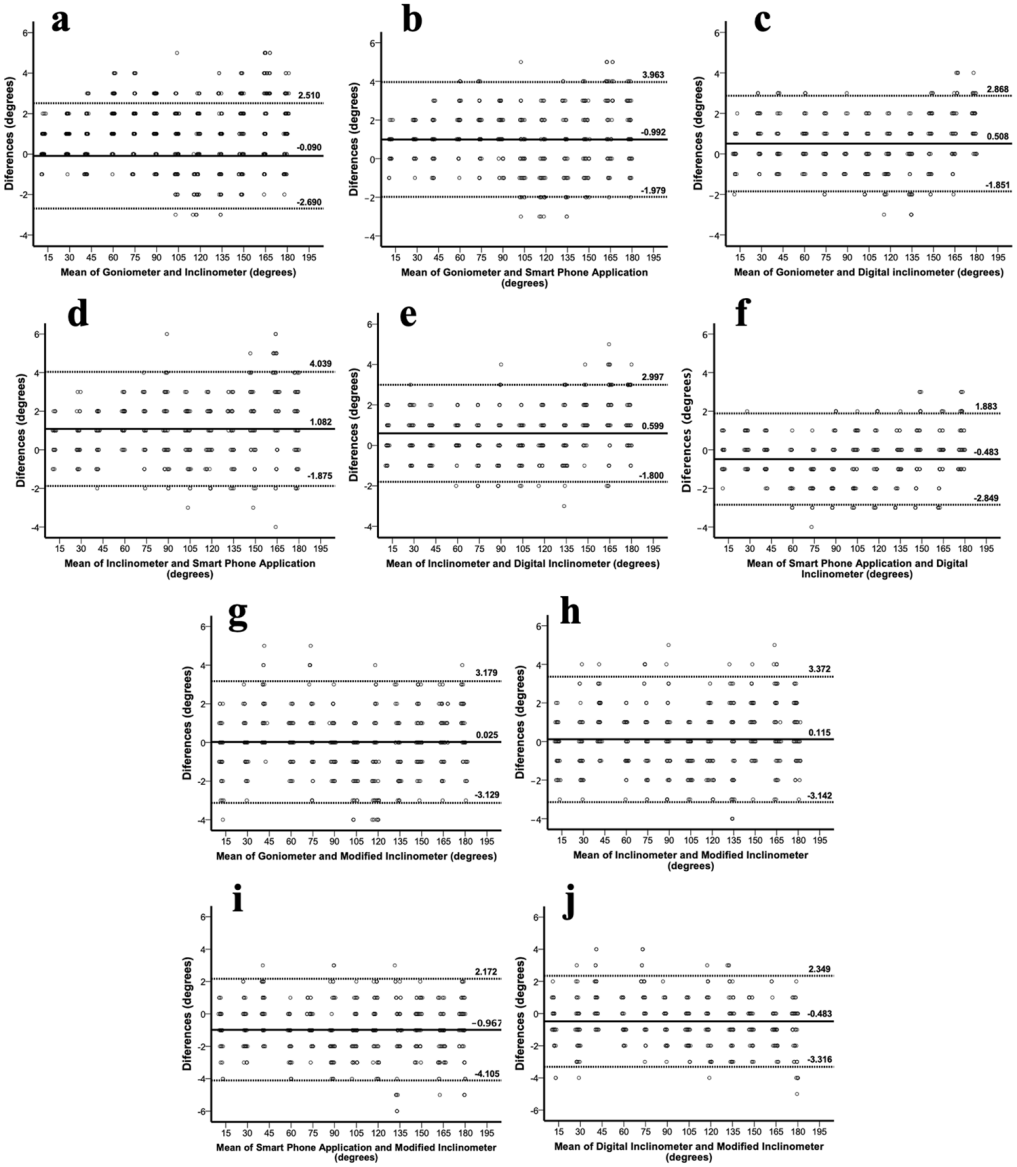
Bland–Altman plots of universal goniometer, inclinometer, digital inclinometer the and smartphone application when measuring standard angle. Bland–Altman plots comparing: (**a**) Universal goniometer versus Inclinometer; (**b**) Universal goniometer versus Smartphone application; (**c**) Universal goniometer versus Digital inclinometer; (**d**) Inclinometer versus Smartphone application; (**e**) Inclinometer versus Digital inclinometer; (**f**) Smartphone application versus Digital inclinometer; (**g**) Universal goniometer versus Modified inclinometer; (**h**) Inclinometer versus Modified Inclinometer; (**i**) Smartphone application versus Modified inclinometer; (**j**) Digital Inclinometer versus Modified inclinometer.

**Figure 5:**
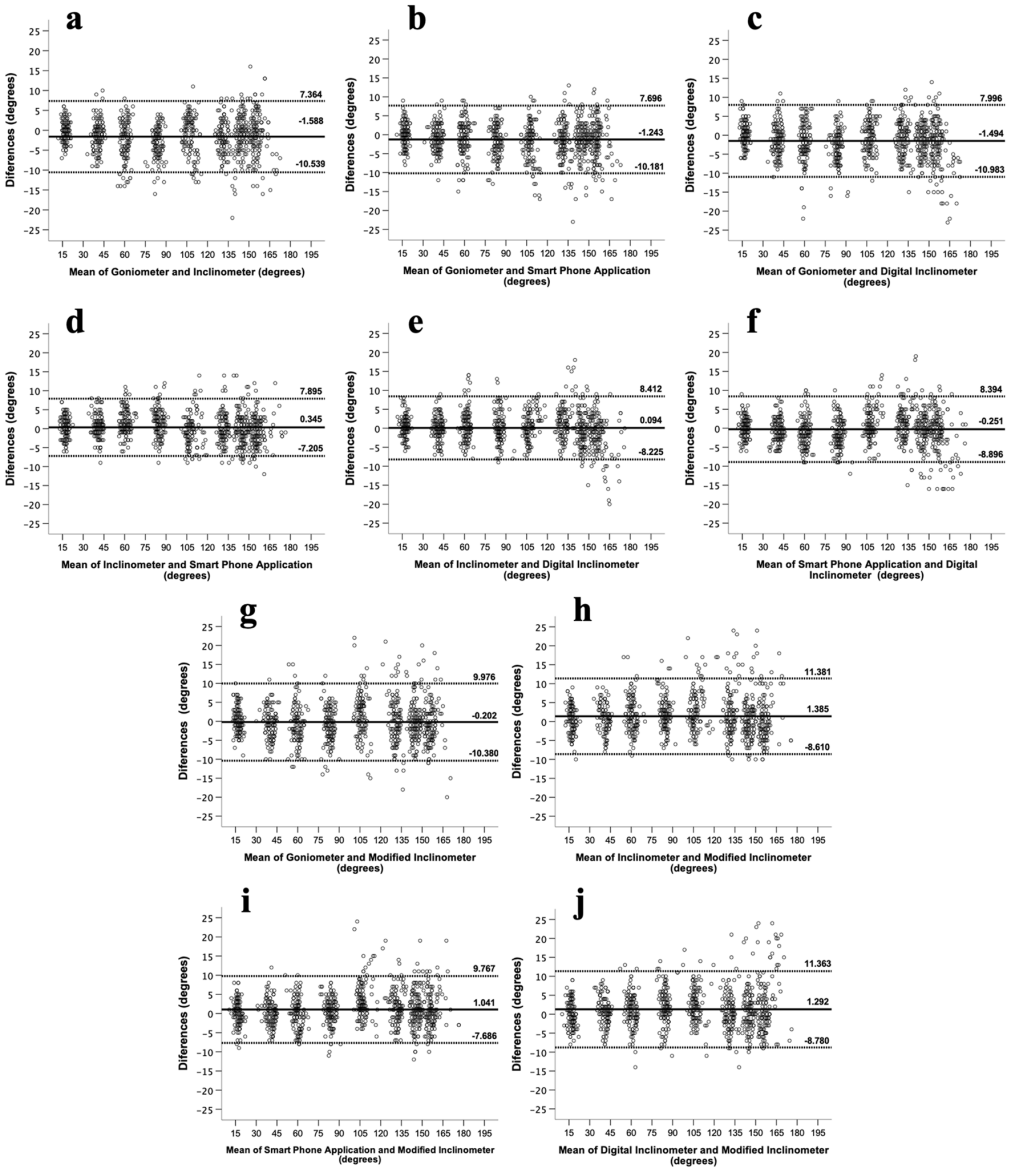
Bland–Altman plots of universal goniometer, inclinometer, digital inclinometer and smartphone application when measuring human shoulder flexion angle. Bland–Altman plots comparing: (**a**) Universal goniometer versus Inclinometer; (**b**) Universal goniometer versus Smartphone application; (**c**) Universal goniometer versus Digital inclinometer; (**d**) Inclinometer versus Smartphone application; (**e**) Inclinometer versus Digital inclinometer; (**f**) Smartphone application versus Digital inclinometer; (**g**) Universal goniometer versus Modified inclinometer; (**h**) Inclinometer versus Modified Inclinometer; (**i**) Smartphone application versus Modified inclinometer; (**j**) Digital Inclinometer versus Modified inclinometer.

In the analysis of concurrent validity, the ICC, SEM, MDC, 95% LOA, and mean of differences between device pairs are shown in Table 4. All device pairs demonstrated ICC values exceeding 0.99 for both standard angle and human joint angle measurements. For measuring standard angles, the three device pairs that included UG, IC, and DI showed an SEM within 1°, MDC within 2°, and 95% LOA between − 2.69° and 3.00°. Device pairs that included SA, MI, and other devices demonstrated a trend toward a greater SEM, MDC, and 95% LOA (0.92°–1.32°, 2°–3°, and − 4.11°–4.04°, respectively). When measuring human joint angles, device pairs that included UG, IC, SA, and DI showed an SEM within 3°, MDC within 8°, and 95% LOA between − 10.98° and 8.41°. In contrast, device pairs that included MI and other devices tended to have higher SEM, MDC, and 95% LOA values (4°, 7°–9°, and − 10.38°–11.38°, respectively). The Bland–Altman plots of each pair, demonstrating their scatter, are shown in Figs. 4 and 5.

**Table 4:** Statistical summary of agreement of all goniometric measurement devices.

Goniometric device comparison	ICC (95%CI)	MD ± SDMD	SEM (deg)	MDC (deg)	95%LOA (deg)
Measure standard angle					
Universal goniometer versus inclinometer	1.000 (1.000, 1.000)	− 0.09 ± 1.33	0.94	2	− 2.69 to 2.51
Universal goniometer versus smartphone application	0.999 (0.998, 1.000)	0.99 ± 1.52	1.28	3	− 1.98 to 3.96
Universal goniometer versus digital inclinometer	1.000 (0.999, 1.000)	0.51 ± 1.20	0.92	2	− 1.85 to 2.87
Universal goniometer versus modified inclinometer	1.000 (0.999, 1.000)	0.03 ± 1.61	1.14	3	− 3.13 to 3.18
Inclinometer versus smartphone application	0.999 (0.998, 1.000)	1.08 ± 1.51	1.31	3	− 1.88 to 4.04
Inclinometer versus digital inclinometer	1.000 (0.999, 1.000)	0.60 ± 1.22	0.96	2	− 1.80 to 3.00
Inclinometer versus modified inclinometer	0.999 (0.999, 1.000)	0.12 ± 1.66	1.18	3	− 3.14 to 3.37
Smartphone application versus digital inclinometer	1.000 (1.000, 1.000)	− 0.48 ± 1.21	0.92	2	− 2.85 to 1.88
Smartphone application versus modified inclinometer	0.999 (0.998, 1.000)	− 0.97 ± 1.60	1.32	3	− 4.11 to 2.17
Digital inclinometer versus modified inclinometer	1.000 (0.999, 1.000)	− 0.48 ± 1.44	1.08	3	− 3.32 to 2.35
Measure human shoulder ROM					
Universal goniometer versus inclinometer	0.995 (0.995, 0.996)	− 1.59 ± 4.57	3	8	− 10.54 to 7.36
Universal goniometer versus smartphone application	0.996 (0.995, 0.996)	− 1.24 ± 4.56	3	7	− 10.18 to 7.70
Universal goniometer versus digital inclinometer	0.995 (0.994, 0.996)	− 1.49 ± 4.84	3	8	− 10.98 to 8.00
Universal goniometer versus modified inclinometer	0.994 (0.993, 0.995)	− 0.20 ± 5.19	4	9	− 10.38 to 9.98
Inclinometer versus smartphone application	0.997 (0.996, 0.997)	0.34 ± 3.85	3	6	− 7.20 to 7.89
Inclinometer versus digital inclinometer	0.996 (0.996, 0.997)	0.09 ± 4.24	3	7	− 8.22 to 8.41
Inclinometer versus modified inclinometer	0.994 (0.994, 0.995)	1.39 ± 5.10	4	9	− 8.61 to 11.38
Smartphone application versus digital inclinometer	0.996 (0.995, 0.996)	− 0.25 ± 4.41	3	7	− 8.90 to 8.39
Smartphone application versus modified inclinometer	0.996 (0.995, 0.996)	1.04 ± 4.45	3	7	− 7.69 to 9.77
Digital inclinometer versus modified inclinometer	0.994 (0.994, 0.995)	1.29 ± 5.14	4	9	− 8.78 to 11.36

*ROM* Range of Motion, *ICC* Intraclass correlation coefficient; *MD* mean of differences, *SDMD* SD of MD, *SEM* standard error of measurement, *MDC* minimal detectable change and *95% LOA* 95% limits of agreement for all goniometric measurement devices.

Interrater analysis for all measurement devices suggested excellent reliability for each standardized angle and the overall ROM (ICC between 0.980 and 0.999). DI showed the lowest SEM (0.61°–1.05°) and MDC (1°–2°) for each standardized angle. UG and IC had an SEM within 1.48° and MDC within 3°. SA and MI showed a trend toward lower reliability, with a greater SEM (0.59°–1.75°) and MDC (1°–4°) than the other devices. All devices, except for DI, tended to have lower interrater reliability (with a greater SEM and MDC) under wider ROM conditions (Table 5). For human joint angle measurement, all measurement devices exhibited varying levels of interrater reliability across all joint angles, ranging from moderate to excellent (ICC between 0.697 and 0.975; SEM between 1.93° and 4.64°; and MDC between 5° and 11°). All devices exhibited lower interrater reliability when measuring wider ROMs, particularly in the fourth quarter of joint angles, showing moderate reliability (ICC between 0.680 and 0.744; SEM between 3.46° and 4.64°; and MDC between 7° and 11°) (Table 5).

**Table 5:** Interrater reliability metrics.

Device	Angle/ROM	1st Measurement			2nd Measurement			Overall		
		ICC (95%CI)	SEM (deg)	MDC (deg)	ICC (95%CI)	SEM (deg)	MDC (deg)	ICC (95%CI)	SEM (deg)	MDC (deg)
Standard angle (n = 30)										
UG	Q1	0.996 (0.992, 0.998)	0.81	2	0.997 (0.994, 0.998)	0.70	2	0.996 (0.994, 0.998)	0.76	2
	Q2	0.991 (0.981, 0.996)	1.10	3	0.997 (0.990, 0.999)	0.68	2	0.994 (0.987, 0.997)	0.92	2
	Q3	0.984 (0.913, 0.994)	1.61	4	0.996 (0.988, 0.998)	0.82	2	0.989 (0.966, 0.995)	1.28	3
	Q4	0.978 (0.827, 0.993)	1.90	4	0.996 (0.992, 0.998)	0.84	2	0.989 (0.966, 0.995)	1.48	3
	Overall	0.999 (0.998, 1.000)	1.43	3	1.000 (1.000, 1.000)	0.76	2	1.000 (0.999, 1.000)	1.15	3
IC	Q1	0.996 (0.992, 0.998)	0.75	2	0.997 (0.995, 0.999)	0.66	2	0.997 (0.995, 0.998)	0.70	2
	Q2	0.990 (0.957, 0.996)	1.21	3	0.996 (0.992, 0.998)	0.75	2	0.993 (0.989, 0.996)	1.01	2
	Q3	0.988 (0.963, 0.995)	1.36	3	0.992 (0.983, 0.996)	1.10	3	0.990 (0.982, 0.994)	1.24	3
	Q4	0.986 (0.948, 0.995)	1.52	4	0.993 (0.980, 0.997)	0.99	2	0.989 (0.972, 0.995)	1.28	3
	Overall	0.999 (0.999, 1.000)	1.25	3	1.000 (1.000, 1.000)	0.89	2	1.000 (0.999, 1.000)	1.08	3
SA	Q1	0.998 (0.996, 0.999)	0.55	1	0.997 (0.993, 0.999)	0.63	1	0.998 (0.995, 0.999)	0.59	1
	Q2	0.993 (0.988, 0.997)	0.99	2	0.987 (0.951, 0.995)	1.40	3	0.990 (0.983, 0.994)	1.21	3
	Q3	0.994 (0.977, 0.998)	0.95	2	0.967 (0.604, 0.991)	2.28	5	0.980 (0.900, 0.992)	1.75	4
	Q4	0.987 (0.948, 0.995)	1.45	3	0.977 (0.924, 0.991)	1.95	5	0.982 (0.926, 0.993)	1.71	4
	Overall	1.000 (0.999, 1.000)	1.04	2	0.999 (0.997, 0.999)	1.70	4	0.999 (0.999,1.000)	1.41	3
DI	Q1	0.998 (0.995, 0.999)	0.61	1	0.997 (0.994, 0.998)	0.72	2	0.997 (0.996, 0.998)	0.67	2
	Q2	0.996 (0.993, 0.998)	0.71	2	0.998 (0.997, 0.999)	0.48	1	0.997 (0.996, 0.998)	0.61	1
	Q3	0.994 (0.985, 0.997)	0.96	2	0.993 (0.982, 0.997)	1.09	3	0.993 (0.988, 0.996)	1.03	2
	Q4	0.991 (0.932, 0.997)	1.19	3	0.995 (0.947, 0.998)	0.88	2	0.993 (0.966, 0.997)	1.05	2
	Overall	1.000 (0.999, 1.000)	0.90	2	1.000 (1.000, 1.000)	0.83	2	1.000 (1.000, 1.000)	0.86	2
MI	Q1	0.989 (0.979, 0.994)	1.25	3	0.997 (0.994, 0.999)	0.67	2	0.993 (0.989, 0.996)	1.01	2
	Q2	0.977 (0.957, 0.988)	1.86	4	0.990 (0.980, 0.995)	1.24	3	0.983 (0.973, 0.989)	1.58	4
	Q3	0.975 (0.916, 0.990)	1.90	4	0.994 (0.978, 0.998)	1.01	2	0.985 (0.973, 0.991)	1.53	4
	Q4	0.974 (0.700, 0.993)	2.06	5	0.990 (0.937, 0.997)	1.23	3	0.982 (0.959, 0.991)	1.71	4
	Overall	0.999 (0.998, 0.999)	1.81	4	1.000 (0.999, 1.000)	1.07	2	0.999 (0.999, 0.999)	1.48	3
Human shoulder ROM (n = 40)										
UG	Q1	0.950 (0.916, 0.972)	2.59	6	0.955 (0.925, 0.975)	2.60	6	0.952 (0.930, 0.968)	2.60	6
	Q2	0.907 (0.830, 0.950)	3.69	9	0.939 (0.896, 0.966)	3.03	7	0.923 (0.877, 0.951)	3.36	8
	Q3	0.924 (0.876, 0.957)	3.52	8	0.943 (0.906, 0.968)	2.98	7	0.933 (0.903, 0.955)	3.25	8
	Q4	0.713 (0.563, 0.826)	3.94	9	0.795 (0.684, 0.877)	2.83	7	0.744 (0.655, 0.818)	3.46	8
	Overall	0.995 (0.993, 0.996)	3.42	8	0.996 (0.995, 0.997)	3.02	7	0.996 (0.995, 0.996)	3.03	7
IC	Q1	0.962 (0.936, 0.979)	2.43	6	0.966 (0.935, 0.982)	2.27	5	0.964 (0.942, 0.977)	2.34	5
	Q2	0.945 (0.909, 0.969)	2.96	7	0.950 (0.911, 0.972)	2.73	6	0.947 (0.921, 0.965)	2.84	7
	Q3	0.880 (0.736, 0.941)	4.80	11	0.915 (0.781, 0.961)	3.76	9	0.895 (0.802, 0.941)	4.35	10
	Q4	0.715 (0.564, 0.828)	4.16	10	0.616 (0.45, 0.756)	4.24	10	0.674 (0.567, 0.765)	4.21	10
	Overall	0.994 (0.992, 0.995)	3.75	9	0.995 (0.993, 0.996)	3.36	8	0.994 (0.993, 0.996)	3.71	9
SA	Q1	0.973 (0.955, 0.985)	1.99	5	0.978 (0.963, 0.987)	1.85	4	0.975 (0.964, 0.983)	1.93	5
	Q2	0.957 (0.929, 0.976)	2.61	6	0.968 (0.947, 0.982)	2.25	5	0.962 (0.946, 0.974)	2.44	6
	Q3	0.901 (0.810, 0.948)	4.12	10	0.916 (0.844, 0.955)	3.70	9	0.907 (0.838, 0.945)	3.92	9
	Q4	0.697 (0.550, 0.813)	4.21	10	0.702 (0.556, 0.817)	3.94	9	0.697 (0.592, 0.784)	4.06	9
	Overall	0.995 (0.994, 0.996)	3.43	8	0.996 (0.995, 0.997)	3.07	7	0.996 (0.994, 0.996)	3.06	7
DI	Q1	0.976 (0.955, 0.987)	1.90	4	0.964 (0.940, 0.980)	2.46	6	0.970 (0.954, 0.980)	2.17	5
	Q2	0.945 (0.909, 0.968)	3.03	7	0.947 (0.909, 0.970)	2.91	7	0.945 (0.921, 0.963)	2.98	7
	Q3	0.923 (0.869, 0.956)	3.52	8	0.948 (0.906, 0.972)	2.86	7	0.934 (0.899, 0.958)	3.23	8
	Q4	0.627 (0.431, 0.774)	5.61	13	0.677 (0.525, 0.800)	3.91	9	0.680 (0.576, 0.768)	4.64	11
	Overall	0.994 (0.991, 0.995)	3.78	9	0.996 (0.994, 0.997)	3.04	7	0.995 (0.993, 0.996)	3.42	8
MI	Q1	0.945 (0.892, 0.971)	2.87	7	0.951 (0.919, 0.972)	2.78	6	0.947 (0.923, 0.965)	2.84	7
	Q2	0.920 (0.868, 0.954)	3.51	8	0.946 (0.911, 0.969)	2.94	7	0.932 (0.903, 0.954)	3.25	8
	Q3	0.924 (0.876, 0.956)	3.91	9	0.906 (0.823, 0.950)	4.14	10	0.914 (0.878, 0.942)	4.05	9
	Q4	0.696 (0.549, 0.813)	3.80	9	0.757 (0.631, 0.852)	3.79	9	0.728 (0.635, 0.805)	3.82	9
	Overall	0.994 (0.993, 0.996)	3.69	9	0.995 (0.993, 0.996)	3.42	8	0.995 (0.993, 0.996)	3.39	8

*ROM* Range of Motion, *ICC* Interclass correlation coefficient, *95%CI* 95% confidence interval, *SEM* standard error of measurement, and *MDC* minimal detectable change for all goniometric measurement devices, *UG* Universal Goniometer, *IC* Inclinometer, *SA* Smartphone Application, *DI* Digital Inclinometer, *MI* Modified Inclinometer, *Q1* 0°–45°, *Q2* > 45°–90°, *Q3* > 90°–135°, *Q4* > 135°–180°.

Analysis of intrarater reliability (Table 6), with each examiner and overall, demonstrated that all devices had excellent reliability (ICC = 0.977 to > 0.999) for each standardized angle and overall ROM. The DI had the lowest SEM and MDC for each standardized angle (0.56°–0.90° and 1°–2°, respectively), whereas MI had the highest SEM and MDC (1.04°–1.91° and 2°–4°, respectively). The UG, IC, and SA had SEM values within 1.43° and MDC values within 3°. All devices, except for DI, tended to have lower intrarater reliability (with greater SEM and MDC values) under wider ROM conditions. For human joint angle measurement, all measurement devices exhibited varying levels of reliability across all joint angles, ranging from moderate to excellent (ICC between 0.660 and 0.996; SEM between 0.77° and 4.06°; and MDC between 2° and 9°). All devices exhibited lower intrarater reliability when measuring wider ROM, particularly in the fourth quarter of joint angles, showing moderate reliability (ICC between 0.660 and 0.842; SEM between 2.95° and 4.06°; and MDC between 7° and 9°).

**Table 6:** Intrarater reliability metrics.

Device	Measure angle	Examiner 1			Examiner 2			Examiner 3			Overall		
		ICC(95%CI)	SEM (deg)	MDC (deg)	ICC(95%CI)	SEM (deg)	MDC (deg)	ICC(95%CI)	SEM (deg)	MDC (deg)	ICC(95%CI)	SEM (deg)	MDC (deg)
Standard angle (n = 30)													
UG	Q1	0.997 (0.994, 0.999)	0.68	2	0.996 (0.990, 0.998)	0.79	2	0.997 (0.988, 0.999)	0.63	1	0.997 (0.994, 0.998)	0.70	2
	Q2	0.993 (0.984, 0.996)	1.05	2	0.995 (0.989, 0.997)	0.87	2	0.997 (0.994, 0.999)	0.62	1	0.995 (0.992, 0.997)	0.86	2
	Q3	0.994 (0.987, 0.997)	0.97	2	0.988 (0.890, 0.996)	1.40	3	0.996 (0.991, 0.998)	0.82	2	0.992 (0.987, 0.995)	1.09	3
	Q4	0.988 (0.767, 0.997)	1.44	3	0.995 (0.986, 0.998)	0.88	2	0.980 (0.541, 0.995)	1.79	4	0.987 (0.967, 0.994)	1.43	3
	Overall	1.000 (0.999, 1.000)	1.07	2	1.000 (0.999, 1.000)	1.02	2	1.000 (0.999, 1.000)	1.09	3	1.000 (0.999, 1.000)	1.06	2
IC	Q1	0.995 (0.941, 0.999)	0.85	2	0.998 (0.995, 0.999)	0.85	2	0.991 (0.678, 0.998)	1.19	3	0.995 (0.963, 0.998)	0.91	2
	Q2	0.991 (0.906, 0.998)	1.12	3	0.994 (0.925, 0.998)	1.16	3	0.995 (0.959, 0.998)	0.85	2	0.993 (0.990, 0.996)	0.99	2
	Q3	0.994 (0.987, 0.997)	0.97	2	0.988 (0.974, 0.994)	0.95	2	0.990 (0.817, 0.998)	1.27	3	0.991 (0.986, 0.994)	1.21	3
	Q4	0.994 (0.986, 0.997)	0.99	2	0.995 (0.989, 0.998)	1.00	2	0.992 (0.960, 0.997)	1.11	3	0.993 (0.990, 0.996)	1.01	2
	Overall	1.000 (0.999, 1.000)	0.99	2	1.000 (0.999, 1.000)	0.98	2	1.000 (0.995, 1.000)	1.12	3	1.000 (0.999, 1.000)	1.03	2
SA	Q1	0.998 (0.990, 0.999)	0.59	1	0.996 (0.959, 0.999)	0.77	2	0.998 (0.993, 0.999)	0.53	1	0.997 (0.987, 0.999)	0.64	1
	Q2	0.990 (0.968, 0.996)	1.27	3	0.994 (0.821, 0.998)	0.94	2	0.994 (0.988, 0.997)	0.91	2	0.992 (0.988, 0.995)	1.05	2
	Q3	0.991 (0.761, 0.998)	1.16	3	0.988 (0.670, 0.997)	1.35	3	0.988 (0.892, 0.996)	1.37	3	0.989 (0.982, 0.993)	1.30	3
	Q4	0.992 (0.599, 0.998)	1.06	2	0.988 (0.967, 0.995)	1.46	3	0.981 (0.123, 0.996)	1.68	4	0.987 (0.838, 0.996)	1.43	3
	Overall	1.000 (0.999, 1.000)	1.06	2	0.999 (0.996, 1.000)	1.17	3	0.999 (0.998, 1.000)	1.21	3	1.000 (0.999, 1.000)	1.15	3
DI	Q1	0.998 (0.991, 0.999)	0.62	1	0.993 (0.966, 0.997)	1.08	3	0.995 (0.857, 0.999)	0.88	2	0.995 (0.971, 0.998)	0.88	2
	Q2	0.998 (0.996, 0.999)	0.53	1	0.999 (0.997, 0.999)	0.45	1	0.997 (0.993, 0.998)	0.68	2	0.998 (0.997, 0.999)	0.56	1
	Q3	0.997 (0.987, 0.999)	0.71	2	0.995 (0.986, 0.998)	0.84	2	0.995 (0.987, 0.998)	0.85	2	0.996 (0.994, 0.997)	0.80	2
	Q4	0.995 (0.985, 0.998)	0.94	2	0.999 (0.997, 0.999)	0.47	1	0.991 (0.302, 0.998)	1.15	3	0.995 (0.984, 0.998)	0.90	2
	Overall	1.000 (1.000, 1.000)	0.72	2	1.000 (1.000, 1.000)	0.76	2	1.000 (0.999, 1.000)	0.91	2	1.000 (1.000, 1.000)	0.80	2
MI	Q1	0.991 (0.963, 0.997)	1.15	3	0.994 (0.988, 0.997)	0.90	2	0.992 (0.969, 0.997)	1.06	2	0.992 (0.981, 0.996)	1.04	2
	Q2	0.990 (0.862, 0.997)	1.23	3	0.981 (0.956, 0.992)	1.68	4	0.979 (0.956, 0.990)	1.79	4	0.983 (0.969, 0.990)	1.59	4
	Q3	0.995 (0.987, 0.998)	0.88	2	0.984 (0.967, 0.993)	1.56	4	0.966 (0.554, 0.991)	2.22	5	0.982 (0.969, 0.989)	1.65	4
	Q4	0.995 (0.984, 0.998)	0.88	2	0.994 (0.988, 0.997)	0.95	2	0.946 (0.007, 0.989)	2.98	7	0.977 (0.927, 0.990)	1.91	4
	Overall	1.000 (0.998, 1.000)	1.05	2	0.999 (0.999, 1.000)	1.32	3	0.998 (0.992, 0.999)	2.16	5	0.999 (0.998, 0.999)	1.58	4
Human shoulder ROM (n = 40)													
UG	Q1	0.966 (0.937, 0.982)	2.23	5	0.959 (0.924, 0.978)	2.44	6	0.964 (0.932, 0.981)	2.20	5	0.962 (0.947, 0.974)	2.31	5
	Q2	0.960 (0.920, 0.980)	2.34	5	0.922 (0.857, 0.958)	3.26	8	0.980 (0.962, 0.989)	1.86	4	0.950 (0.929, 0.965)	2.71	6
	Q3	0.918 (0.851, 0.956)	3.82	9	0.898 (0.811, 0.945)	3.97	9	0.955 (0.916, 0.976)	2.56	6	0.928 (0.899, 0.950)	3.36	8
	Q4	0.698 (0.309, 0.859)	3.85	9	0.531 (0.266, 0.721)	5.11	12	0.826 (0.697, 0.904)	2.47	6	0.660 (0.546, 0.750)	3.98	9
	Overall	0.994 (0.984, 0.997)	3.67	9	0.993 (0.991, 0.995)	4.08	10	0.998 (0.997, 0.998)	2.14	5	0.996 (0.995, 0.996)	3.03	7
IC	Q1	0.976 (0.955, 0.987)	1.86	4	0.975 (0.953, 0.987)	1.97	5	0.988 (0.977, 0.993)	1.39	3	0.978 (0.968, 0.985)	1.83	4
	Q2	0.964 (0.934, 0.981)	2.48	6	0.942 (0.893, 0.969)	2.79	7	0.962 (0.929, 0.980)	2.44	6	0.956 (0.937, 0.969)	2.58	6
	Q3	0.918 (0.851, 0.956)	4.12	10	0.962 (0.929, 0.980)	2.50	6	0.965 (0.935, 0.981)	2.41	6	0.935 (0.908, 0.954)	3.43	8
	Q4	0.776 (0.615, 0.875)	3.64	8	0.832 (0.705, 0.907)	3.35	8	0.852 (0.738, 0.919)	2.29	5	0.806 (0.733, 0.861)	3.24	8
	Overall	0.995 (0.993, 0.997)	3.43	8	0.997 (0.996, 0.998)	2.65	6	0.998 (0.997, 0.998)	2.10	5	0.996 (0.996, 0.997)	3.03	7
SA	Q1	0.969 (0.942, 0.983)	2.18	5	0.963 (0.932, 0.980)	2.33	5	0.975 (0.954, 0.987)	1.96	5	0.969 (0.955, 0.978)	0.77	2
	Q2	0.979 (0.961, 0.989)	1.90	4	0.945 (0.899, 0.971)	2.76	6	0.972 (0.948, 0.985)	2.16	5	0.964 (0.948, 0.975)	2.20	5
	Q3	0.939 (0.887, 0.967)	3.39	8	0.924 (0.861, 0.959)	3.53	8	0.977 (0.957, 0.988)	1.86	4	0.945 (0.922, 0.962)	3.03	7
	Q4	0.892 (0.805, 0.941)	2.53	6	0.799 (0.650, 0.888)	3.87	9	0.851 (0.735, 0.918)	2.18	5	0.842 (0.780, 0.887)	2.95	7
	Overall	0.997 (0.996, 0.998)	2.67	6	0.996 (0.994, 0.997)	3.09	7	0.998 (0.998, 0.999)	2.14	5	0.997 (0.996, 0.998)	2.65	6
DI	Q1	0.973 (0.949, 0.986)	2.07	5	0.965 (0.934, 0.982)	2.29	5	0.980 (0.963, 0.989)	1.84	4	0.973 (0.961, 0.981)	2.06	5
	Q2	0.973 (0.950, 0.986)	2.12	5	0.920 (0.855, 0.957)	3.70	9	0.971 (0.946, 0.984)	2.10	5	0.952 (0.932, 0.966)	2.78	6
	Q3	0.940 (0.891, 0.968)	3.14	7	0.926 (0.863, 0.961)	3.34	8	0.966 (0.936, 0.982)	2.36	5	0.945 (0.922, 0.962)	2.94	7
	Q4	0.727 (0.539, 0.845)	4.03	9	0.785 (0.628, 0.880)	4.54	11	0.733 (0.551, 0.849)	3.38	8	0.757 (0.668, 0.824)	4.06	9
	Overall	0.996 (0.995, 0.997)	3.05	7	0.995 (0.993, 0.996)	3.49	8	0.997 (0.996, 0.998)	2.61	6	0.996 (0.995, 0.997)	3.06	7
MI	Q1	0.935 (0.878, 0.965)	3.16	7	0.957 (0.921, 0.977)	2.54	6	0.966 (0.931, 0.982)	2.32	5	0.952 (0.932, 0.966)	2.70	6
	Q2	0.917 (0.849, 0.955)	3.67	9	0.918 (0.850, 0.955)	3.55	8	0.973 (0.948, 0.986)	2.05	5	0.952 (0.932, 0.966)	2.73	6
	Q3	0.935 (0.881, 0.965)	3.55	8	0.890 (0.801, 0.940)	4.60	11	0.976 (0.955, 0.987)	2.12	5	0.925 (0.895, 0.947)	3.77	9
	Q4	0.843 (0.557, 0.933)	2.87	7	0.656 (0.423, 0.805)	4.78	11	0.871 (0.768, 0.930)	2.31	5	0.724 (0.572, 0.819)	3.83	9
	Overall	0.995 (0.994, 0.997)	3.41	8	0.992 (0.989, 0.994)	4.30	10	0.998 (0.997, 0.998)	2.12	5	0.995 (0.994, 0.996)	3.38	8

*ROM* Range of Motion, *ICC* Interclass correlation coefficient, *95%CI* 95% confidence interval, *SEM* standard error of measurement, and *MDC* minimal detectable change for all goniometric measurement devices, *UG* Universal Goniometer, *IC* Inclinometer, *SA* Smartphone Application, *DI* Digital Inclinometer, *MI* Modified Inclinometer, *Q1* 0°–45°, *Q2* > 45°–90°, *Q3* > 90°–135°, *Q4* > 135°–180°.

## Discussion

The present study is the first to explore measurement errors, considering both device and examiner factors, with and without human factors. We conducted a thorough examination of measurement error using five goniometric devices, covering a range of available ROM from 0° to 180° across 12 standard measurement angles and 8 human shoulder joint flexion angles. Our findings can serve as reference values for the development of goniometric equipment, both before and after conducting studies on human joints, while also considering errors from the equipment and examiner objectives.

As a primary objective, we conducted a detailed assessment of concurrent validity to investigate the impact of technology-based device designs on examiner performance. We compared four common measurement devices with UG, a standard clinical tool, across two phases: standard angle measurements and human joint angle measurements. Our analysis in both phases yielded ICCs values exceeding 0.99 for all device pairs, demonstrating their reasonable validity^35^. Additionally, the Bland–Altman plots for each device pair displayed even dispersion along the x-axis, with mean differences ranging from − 0.97 to 1.08 for the standard angle measurement phase and − 1.59–1.39 for the human joint angle measurement phase. These results suggest that the differences between the two instruments are consistent and not significantly different^39^. In the standard angle measurement phase, our findings indicated consistency among each device pair. This aligns with the findings of prior research, confirming the potential of technology-based devices to replace UG without introducing significant variability^19,40–42^. However, when measuring human joint angles, notable discrepancies among the devices were observed, highlighting the substantial influence of technology-based device designs on examiner performance in complex scenarios. Of particular significance, both SA and DI stood out because of their utilization of higher-precision embedded technology, which eliminates the need for examiners to read scales or maintain a final position for scale reading. This unique feature sets them apart from traditional measurement tools, such as IC and MI, significantly contributing to their superior performance in measuring human shoulder joint angles^18,41,43^. In conclusion, our study highlights the potential of technology-based devices, particularly SA and DI, in replacing UG and improving measurement accuracy, particularly in complex scenarios, such as measuring human shoulder joint angles. These findings underscore the critical role of device technology and design in examiner performance. To enhance precision and reliability in clinical measurements, considering these factors when selecting tools is crucial. Additionally, our insights suggest that the development of new goniometric devices with features eliminating the need for reading scales or allowing for fixed final scores for later reading could substantially reduce measurement errors in various research and clinical settings.

For the secondary objective, our reliability analysis consistently revealed excellent inter- and intrarater reliabilities (ICC > 0.90) for all standardized angles and the first three quadrants of the human shoulder flexion angle. However, in the last quadrant, reliability ranged from moderate to good levels, for both intra- and interrater assessments^35^. We consistently observed an increasing trend in both intra- and interrater reliabilities as the measurement angles widened. This trend remained consistent across both phases for all devices, except for DI, when measuring standard angles. Notably, this trend became more pronounced when measuring human joint angles. This aligns with the common understanding that measuring human joint angles involves a complex interplay of factors, including device, examiner, and individual-specific factors, resulting in greater measurement variability. These findings parallel the outcomes of our concurrent validity study, which revealed larger and more dispersed mean differences among device pairs at wider angles, particularly in the fourth quadrant of human joint angles. Studies have typically focused on measuring the entire ROM for each joint direction. Note that although the study by Handcook (2018)^8^, a frequently cited literature source, measured three angles of the knee joint, it did not report reliability values for each angle separately. This divergence poses a challenge when comparing our findings with those of previous studies. Furthermore, Wellmon et al. (2016)^19^ explored interrater reliability for standardized acute, right, and obtuse angles. They reported differences in means for measurements performed using SA, suggesting the potential for clinically meaningful differences to arise when measuring angles > 90°, although they could not provide further clarification. Our results support the findings of Wellmon et al., as four of the goniometric devices exhibited the same trend, except for DI. This trend can be attributed to the alignment of the goniometric device’s reference part. Notably, we observed that the reference part tended to shift more when the final position significantly deviated from the starting position. This shift primarily occurred due to substantial alterations in soft tissue tension during closely end-range motion, causing changes in arm shape and consequently affecting reference part alignment. It is imperative to highlight that our study uniquely addressed reliability at various joint angles, encompassing both standardized and human joint angles. However, this trend was not observed when using DI to measure all standard angles. This deviation may be because of the scale-free reading function and the wider width of the DI reference base, which makes it easier to align by placing it on the surface of the apparatus arms at all angles. The clinical implications of this finding suggest that when measuring joint angles across a wide range, it is critical to reconfirm reference part alignment for consistency with the starting position, particularly during significant posture changes in end-range motion. These insights hold promise for enhancing the accuracy and reliability of joint angle measurements in clinical and research applications, aligning with the goal of accurately reflecting clinical changes, such as treatment effectiveness or the progression of a condition.

No previous study has reported reference values for instrument-focused measurement error corresponding to common goniometric devices (ICC of inter- and intrarater reliabilities, concurrent validity, SEM, MDC, and 95% LOA). Such reference values are necessary for non-experimental studies on the development of novel prototype goniometric devices. Our report concurred substantially with the findings of previous studies. Chapleau et al.^23^ examined the reliability and validity of UG compared with those of radiography for ROM measurement of healthy elbows. Regarding concurrent validity, a 95% LOA of ± 10.3 (or less) was reported. The ICC for the interrater reliability of UG ranged from 0.95 to 0.97. Wellmon et al.^19^ studied concurrent validity and reliability by focusing on device and examiner factors and excluded patient factors. They reported an ICC of 0.999 for the concurrent validity of UG and IC, with a 95%LOA ranging from − 3.8 to 3.5. The interrater reliability of UG and IC was also excellent (ICC > 0.99). Hancock et al.^8^ examined the accuracy and reliability of five knee goniometric methods by supporting the limb to maintain knee angles during measurement. They reported excellent intrarater (ICC > 0.98) and interrater (ICC > 0.99) reliabilities, with the minimum significant differences ranging from 6° to 14°, for both short- and long-arm and laser projection-based digital goniometers. Kolber and Hanney^30^ reported the interrater reliability of IC for identifying posterior shoulder tightness. Excellent reliability (ICC = 0.90) with an MDC of 9° and SEM within 4° was reported. UG, IC, and DI are commonly used in clinical practice^16^ and have been recommended as the gold standard by numerous studies^8,18,19,29,31,44^. Therefore, measurement error metrics based on these three devices can be recommended as reference values. In the light of our findings, it can be concluded that ICC values for inter- and intrarater reliabilities should be > 0.90, SEM should not exceed 2°, and MDC should not be greater than 3°. In terms of concurrent validity, UG and IC set the reference device; ICC values should be > 0.90, SEM should not be greater than 1°, and 95% LOA should range from − 3° to 3°; these criteria can set the error limits for measuring standardized joint angles in non-experimental studies of goniometer prototypes.

In the development of new goniometric devices, extending accuracy testing to include human joint measurements after assessing known angles is essential. This is important because of the variability among individuals, which can have a substantial impact on both the device’s performance and the examiner’s accuracy. Our findings *showed that wider joint angles led to increased measurement errors, especially in human joint measurements. This is* because of the complex interplay of factors, including tissue tension, changes in limb shape, and misalignment from the starting position. Considering these factors and the specific characteristics of each device, we must analyze the sources of measurement error, discuss control methods and furthermore make recommendations for developing more accurate clinical goniometric devices. Incorporating considerations of intra- and interrater reliability and concurrent validity in human joint measurements from our findings is crucial.

UG demands a high level of examiner skill and involves scale reading, although it does not require holding the final position for immediate scale reading (the final score can be fixed and read later). It necessitates aligning three anatomy points: the axis and both the stationary and movable arms, which places a premium on detailed anatomical identification. However, this feature is advantageous when realigning the zero-starting position upon reaching the final ending position. Although this characteristic presents minimal challenges when measuring standard angles because of their clear and easily definable axes and arms, it poses difficulties in measuring human joints, particularly in large joint angle quadrants where defining the axis and reference body parts becomes more intricate. Although, the fluid level inclinometer in this study requires scale reading and stabilization of the final position for scale reading. Nevertheless, it has a short reference base, which is contrary to previous studies that indentified the positive effect of extending the goniometer arm on measurement accuracy^8,45^.

The DI demonstrated superior validity and intra- and interrater reliabilities when used to measure standard angles. However, it did not exhibit the same level of superiority when measuring human joints. The DI wide reference base width facilitated its deployment on standard angle arms but did not yield a similar positive effect when measuring human joints. On the other hand, the DI short reference base and large size made alignment more challenging, particularly when measuring human joints near the end range. Contrary to previous research findings^27,28^, our study showed that the DI's reliability for ROM assessment was lower compared to the UG. However, our findings provided greater validity and reliability than those of Kolber et al.^18^, who examined the reliability and concurrent validity of shoulder mobility measurements using a DI compared with those of shoulder mobility measurements using a UG. They reported an SEM of 2° and a LOA of ± 11°, which are reasonable values for patient measurements. Our MDC values also achieved improved accuracy relative to those reported by Mohammad et al.^29^, who noted MDC values ranging from 1.45° to 11.89° when assessing ROM in lower extremity joints. A direct comparison is however challenging due to differences in angle sources, study populations, and the use of a different specific model of the DI.

When measuring standardized joint angles, MI exhibited higher measurement errors than the other devices. However, their ICC values for concurrent validity exceeded 0.90, indicating excellent interrater and intrarater reliabilities, which are generally considered acceptable^35^. MI exhibited slightly inferior concurrent validity and intra- and interrater reliabilities when measuring both standardized and human joint angles. This could be attributed to the need for scale reading and holding the final position for scale reading. In contrast, MI only required an initial reference setting (zero starting) and then reading the scale at the final position, which limits adjustments to the final alignment. Additionally, this difference in performance might be related to partially unstable fixation between MI and the measurement apparatus. Body shape changes occur beneath the fixing apparatus because of the tension of the surrounding soft tissue. This differs from that shown in a previous study that measured neck movement and applied a fixing apparatus (tape) around the head, where there was less significant shape change during measurement^42^. Clinically, applying the fixing apparatus to areas with minimal shape changes, such as bony prominences, is advisable to ensure more stable measurements.

For standardized angle measurements, SA showed slightly decreased reliability, which is consistent with the findings of prior research highlighting design-related variability, particularly due to rounded edges. This finding agrees with that reported by Wellmon et al.^19^, who investigated the concurrent validity and interrater reliability of the Goniometer Record and Goniometer Pro applications installed on various smartphones for measuring standardized angles. They considered UG and IC as the reference standards. Their study revealed ICC values for concurrent validity (using both applications) exceeding 0.99 and 95% LOA within ± 4.05°, indicating strong agreement. Interrater reliability was excellent, with an ICC exceeding 0.99. They emphasized the influence of smartphone design on reliability, particularly when placing the smartphone’s edge against a flat testing apparatus surface. When measuring human joint angles, SA exhibited excellent concurrent validity, with an ICC exceeding 0.90, SEM within 3°, MDC within 7°, and 95% LOA ranging from ± 10°. Furthermore, it demonstrated impressive intrarater reliability, with an ICC exceeding 0.90, SEM within 4°, and MDC within 7°, and strong interrater reliability, featuring an ICC exceeding 0.90, SEM within 5°, and MDC within 9°. These findings in the present human study highlight the superior validity and reliability of SA compared with those of other devices. This can be attributed to the high-precision technology embedded^46^ and the technique employed, which aligns smartphone reference lines with humerus positioning, effectively mitigating variations caused by nonflat surfaces. Several factors likely contributed to these excellent results, including the absence of scale reading, the capability to establish references twice (initially at the zero starting position and later at the final position, with the option to adjust alignment in both instances), and the extended length of the smartphone's edge (long side), which enhanced alignment with the humerus^2^.

In a study by Ockendon and Gilbert^47^, the validity of a novel smartphone accelerometer-based goniometer was assessed, examining 5°–45° of knee flexion deformity compared with a standard Lafayette goniometer. They reported that 95% LOA was ± 7.6°, indicating good agreement. However, earlier studies^48,49^ have reported varying levels of validity and reliability when using Android and iPhone applications to measure cervical ROM among healthy participants, ranging from poor to excellent. Chapeau et al.^23^ conducted a noteworthy study on radiographic elbow measurements, reporting interrater ICC values ranging from 0.98 to 0.99. They recommended that a clinically acceptable maximal measurement error should not exceed 10°. In conclusion, both a gyroscope-based smartphone application (using the Goniometer Records application) and a modified gravity pendulum inclinometer (IC) with a fixing apparatus proved suitable for measuring the feasible range of motion in clinical practice. However, when developing new clinical goniometric devices aimed at challenging validity and reliability, SA should be considered a reference device with its unique set of challenges under human joint testing phase.

The limitation of our study is that it focused solely on measuring shoulder flexion in one direction. Future studies should consider measuring motion angles in other directions and examining joints with pathological conditions. Additionally, following this, elegant finite element studies may be conducted using the data extracted to assist in developing clinically more accurate numerical simulations for bioengineering.

## Conclusions

Our study provides insights into the capabilities of three examiners to accurately use five commonly used clinical goniometers (i.e., UG, IC, SA, DI, and MI), focusing on device and examiner factors, considering their impact with and without human-specific factors in order to derive reference values for error quantification and clarify what objective applies when developing a new device for measuring ROM. Testing should start with an examination of known standard angles. We recommend that the ICC of reliability should be greater than 0.90, the SEM should be less than 2°, and the MDC should not be greater than 3°. The most accurate and reliable goniometric measurement devices, in terms of all error metrics, were DI for standardized angle measurements and SA for human joint angle measurements. When developing a new clinical goniometric device and challenging its validity and reliability, DI and SA should be considered as reference devices for testing standardized angles and human joint angles, respectively. For standardized joint angles, concurrent validity should meet the criteria of ICC greater than 0.90, SEM less than 1°, MDC within 2°, and 95% LOA within ± 3°. For human joint angles, concurrent validity should adhere to the criteria of ICC greater than 0.90, SEM less than 3°, MDC within 7°, and 95% LOA within ± 10°. Factors, such as the absence of scale reading, the inclusion of a fixing final scale function and ensuring a sufficiently long reference part may play crucial roles. Moreover, we found dissimilar inter- and intrarater reliabilities with varying ROM measurements. We suggest that the concurrent validity and reliability of goniometric prototypes should be studied using all available ROM measurements.

## Data Availability

The datasets used and/or analysed during the current study are available from the corresponding author on reasonable request.
